# Streamlining the Transition From Yeast Surface Display of Antibody Fragment Immune Libraries to the Production as IgG Format in Mammalian Cells

**DOI:** 10.3389/fbioe.2022.794389

**Published:** 2022-05-10

**Authors:** David Fiebig, Jan P. Bogen, Stefania C. Carrara, Lukas Deweid, Stefan Zielonka, Julius Grzeschik, Björn Hock, Harald Kolmar

**Affiliations:** ^1^ Institute for Organic Chemistry and Biochemistry, Technical University of Darmstadt, Darmstadt, Germany; ^2^ Ferring Darmstadt Laboratories, Darmstadt, Germany; ^3^ Ferring Biologics Innovation Centre, Epalinges, Switzerland; ^4^ Centre for Synthetic Biology, Technical University of Darmstadt, Darmstadt, Germany

**Keywords:** antibody hit discovery, bidirectional promoter, reformatting, golden gate cloning, monoclonal antibodies, yeast surface display

## Abstract

Yeast-surface display (YSD) is commonly applied to screen Fab immune or naïve libraries for binders of predefined target molecules. However, reformatting of isolated variants represents a time-intensive bottleneck. Herein, we present a novel approach to facilitate a lean transition from antibody screening using YSD Fab libraries to the production of full-length IgG antibodies in Expi293-F cells. In this study, utilizing Golden Gate Cloning (GGC) and a bidirectional promoter system, an exemplary Fab-displaying YSD library was generated based on immunised transgene rats. After subsequent screening for antigen-specific antibody candidates by fluorescence-activated cell sorting (FACS), the Fab-encoding genes were subcloned into a bidirectional mammalian expression vector, exhibiting CH2-CH3 encoding genes, in a GGC-mediated, PCR-free manner. This novel, straightforward and time-saving workflow allows the VH/VL pairing to be preserved. This study resulted in antibody variants exhibiting suitable biophysical properties and covered a broad VH diversity after two rounds of FACS screening, as revealed by NGS analysis. Ultimately, we demonstrate that the implication of such a gene transfer system streamlines antibody hit discovery efforts, allowing the faster characterisation of antibodies against a plethora of targets that may lead to new therapeutic agents.

## Introduction

Monoclonal antibodies (mAbs) have shown great potential both as therapeutic and diagnostic tools, with the global monoclonal antibody market expected to reach $300 billion in revenues by 2025 (Lu et al., 2020). Today, a wide variety of display technologies are established for the identification of mAb candidates from immune, synthetic or naïve libraries, among them phage display (McCafferty et al., 1990), ribosome display (Schaffitzel et al., 1999; Lipovsek and Plückthun, 2004), mRNA display (Lipovsek and Plückthun, 2004; Josephson et al., 2014), mammalian display (Parthiban et al., 2019) and yeast display (Boder and Wittrup, 1997). However, all these technologies require laborious subcloning of isolated mAb-encoding genes into protein expression vectors. Even though this process was improved within the last years, PCR-based subcloning always bears the risk of incorporating unintended mutations. Due to the increasing interest in developing mAbs against a plethora of targets, we sought out to streamline the antibody hit discovery workflow.

Besides phage display, particularly yeast-surface display (YSD) has become widely applicable for screening of large libraries (Boder and Wittrup, 1997). The first approved therapeutic antibody generated *via* YSD was Sintilimab, a PD-1 blocking antibody, approved in 2018 for the treatment of relapsed or refractory classical Hodgkin’s lymphoma in China (Hoy, 2019; Valldorf et al., 2021). While advances in YSD technology have facilitated the generation of large Fab antibody libraries using streamlined approaches (Rosowski et al., 2018; Roth et al., 2018), the pitfall that follows antibody screening, namely reformation of Fabs into full-length IgG molecules, remains a tedious procedure. Reformatting into IgG molecules is required in order to fully discover the activity and function of mAbs and to assay their properties, such as Fc-mediated functions (Kapur et al., 2014; Bournazos and Ravetch, 2017). Furthermore, the handling of each antibody individually is required in order to preserve the unique VH and VL pairing.

In recent years, Cruz-Teran and others (2017) have shown that a modification of the yeast cell surface allows one to switch between cell-surface display and secretion of full-length antibodies in order to circumvent subcloning of hit candidates into a suitable expression vector for mammalian expression (Cruz-Teran et al., 2017; Krah et al., 2020). Nevertheless, the glycosylation patterns in baker’s yeast cells differ significantly from those in humans (Tanner and Lehle, 1987; Wildt and Gerngross, 2005) and the yields by application of such methods are very limited. On the contrary, two mammalian cell lines are commonly used for small- to mid-scale antibody production due to their human-like glycosylation and high titres, namely Human Embryonic Kidney 293 (HEK293) and Chinese Hamster Ovary (CHO) cells (Li et al., 2010; Vazquez-Lombardi et al., 2018; Carrara et al., 2021a). To continue the production of IgG molecules in mammalian cells and avoid the cumbersome reformatting steps, we have developed a novel two-pot, two-step cloning procedure in order to facilitate the transition of hit candidates from a YSD-display vector to a mammalian bidirectional (BiDi) expression vector. Initial studies were carried out to analyse the most suitable BiDi promoter for both κ- and λ-isotype antibodies (Carrara et al., 2021b). On top of simplifying and facilitating the transition between display on yeast cells to production in mammalian cells, VH and VL pairing is also preserved. To date, a few high-throughput platforms have been described in order to batch reformat from the scFv format to IgG molecules from phage display libraries (Renaut et al., 2012; Batonick et al., 2016; Xiao et al., 2017; Liu et al., 2018; Reader et al., 2019), but to the best of our knowledge, there have been no such reports for YSD Fab libraries.

**FIGURE 1:**
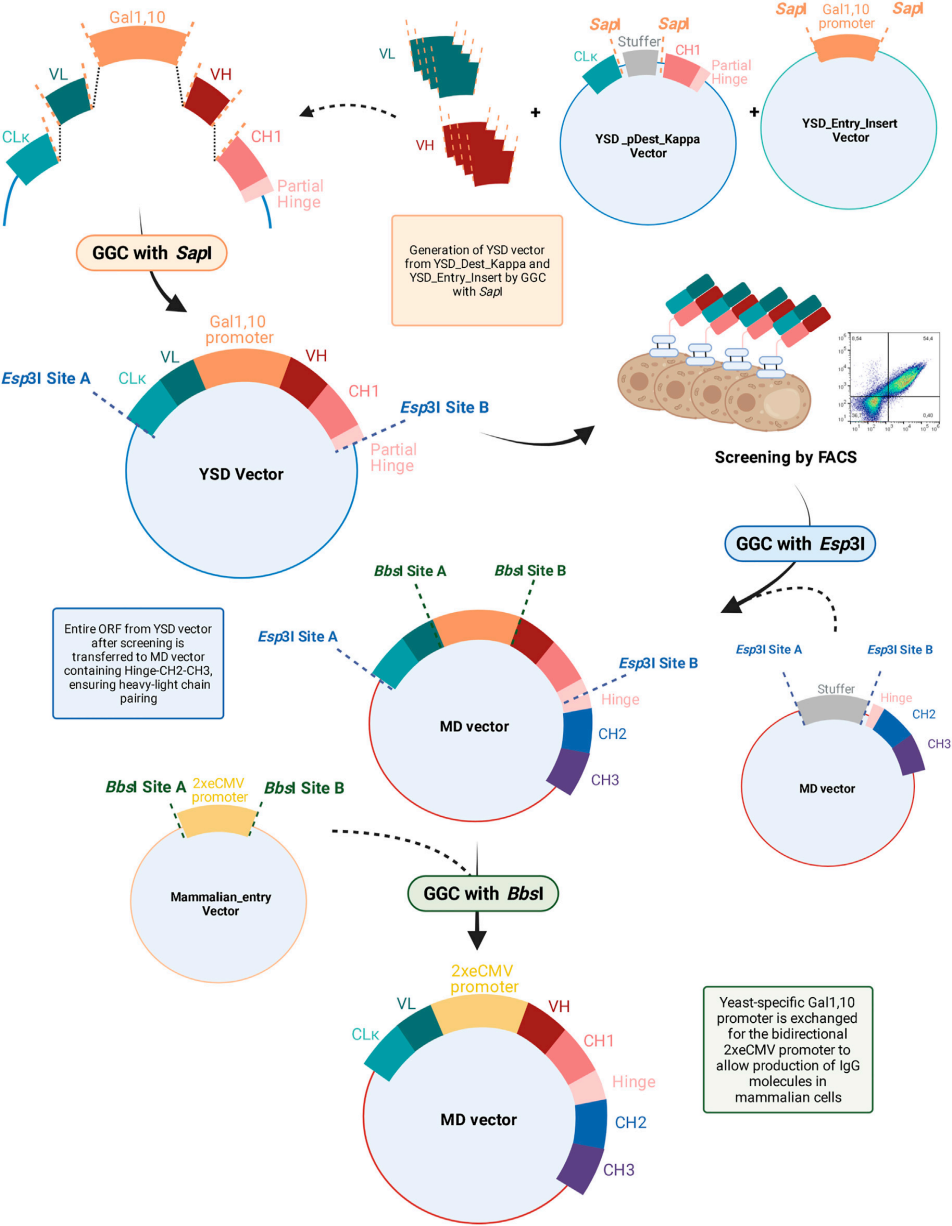
Overview of bulk reformatting workflow from YSD vector to the engineered mammalian destination (MD) vector. VH, heavy chain variable domain; VL, light chain variable domain; CLκ, light chain constant kappa domain; GGC, Golden Gate Cloning; YSD, yeast surface display; MD, mammalian destination; ORF, open reading frame; FACS, fluorescence-activated cell sorting.

In this study, we performed an initial proof-of-concept (PoC) experiment with the therapeutic anti-PD-L1 antibody Durvalumab to establish the reformatting workflow (Figure 1) (Alvarez-Argote and Dasanu, 2019). Subsequently, a Fab library resulting from immunised OmniRats against a TAMR was generated, screened, reformatted, and produced as well as purified using this streamlined approach. The TAM receptors (TAMR), comprising Tyro3, Axl, and MerTK, belong to the family of receptor tyrosine kinases, which have been the focus of several studies over the last decade due to their implications in a number of diseases (Alvarez-Argote and Dasanu, 2019; Reader et al., 2019). The resulting variants unveiled appropriate biophysical properties and covered the entire VH diversity, as revealed by NGS analysis. This method paves the way for facilitating hit discovery processes by expediting the transition between YSD-vectors and mammalian expression of hit candidates.

## Materials and Methods

### Plasmids and Yeast Strains

Plasmids and yeast strains used, as well as their cultivation and media, were previously described in detail elsewhere (Bogen et al., 2020a; Bogen et al., 2020b). For library generation, the yeast destination vector, YSD_pDest_Kappa, comprising a coding sequence for a κ CL and a CH1-hinge-Aga2p fusion, as well as the yeast entry vector, YSD_Entry_Insert, encoding the Gal1,10 promoter were used. By *Sap*I-mediated GGC, VH and VL sequences, previously amplified from cDNA (see below) were subcloned into the destination vector, similar to the approach described previously (Rosowski et al., 2018). The mammalian destination (MD) vector was recently described in detail (Carrara et al., 2021b). In brief, the MD vector encodes a stuffer sequence flanked by *Esp3*I sites, adjacent to coding sequences comprising partial hinge-CH2-CH3 domains. By means of Golden Gate cloning, VL-CL and VH-CH1 encoding sequences can be inserted into MD. Additionally, the bidirectional promoter is flanked by *Bbs*I sites and can be chosen freely. The 2xeCMV promoter combination was shown to be the most suitable for recombinant antibody production and was thus used herein. The sequence of this promoter construct is available from (Carrara et al., 2021b).

### Immunisation

OmniRats (Osborn et al., 2013) were immunised by genetic immunisation encoding TAMR at Aldevron (Freiburg, Germany). After adequate titres were observed, lymph nodes and spleens were extracted. All procedures were carried out in accordance with local animal welfare guidelines and protection laws.

### Library Generation

From immunised OmniRats, total RNA was isolated from lymph nodes and spleens using RNeasy Plus Mini Kit (QIAGEN). Subsequently, cDNA was synthesised using SuperScript III First-Strand Kit (Thermo Fisher Scientific) from 25 µl RNA with random hexamer primers, dNTPs and water to an end volume of 35 µl. The mixture was first incubated at 65°C for 5 min followed by a 1 min incubation on ice. 35 µl containing 1x RT-Buffer, MgCl_2_, DTT, RNase OUT and 200 U SuperScript III RT were added. The mixture was incubated for 5 min at 25°C and 60 min at 50°C. The reaction was terminated by incubating for 5 min at 80°C. 1 µl RNase H was added to each tube and incubated for 20 min at 37°C.

With the cDNA, human antibody variable regions from OmniRats were amplified using two successive, nested PCR reactions. For the first PCR, unique forward primers annealing to the leader sequence of VH or VL kappa were combined with reverse primers annealing to rat CH1 or CL domains, respectively, using Q5-High Fidelity DNA Polymerase. After PCR Clean-Up using Wizard SV Gel and PCR Clean-Up System (Promega) and verifying successful amplification by agarose gel electrophoresis, a second PCR was performed to amplify human VH and VL domains and incorporating *Sap*I recognition sequences for subsequent Golden Gate Cloning (GGC) into the YSD vector (primer sequences can be found in Supplementary Table S1). Therefore, the purified VH and VL PCR products were assembled with the plasmids YSD_Entry_Insert and YSD_pDest_Kappa. For comparison, an additional anti-TAMR Fab library was generated by homologous recombination as described by Benatuil et al. in EBY100 yeast cells (Benatuil et al., 2010).

### Library Sorting

For library sorting, 5 × 10^8^ induced yeast cells were washed with PBS containing 0.1% BSA (PBS-B) and incubated with 250 nM TAMR-His_6_ for 30 min on ice. After washing with PBS-B, cells were stained with Goat F(ab’)_2_-anti-human-Kappa PE-conjugate (1:75 dilution, Southern Biotech) to detect surface presentation, with anti-His6 AlexaFluor647-conjugate (1:50 dilution, Thermo Fisher Scientific) for target binding and incubated for 15 min on ice. After a final washing step, cells were analysed by FACS using a Sony Cell Sorter SH800S. Sorting rounds were performed with decreasing target antigen concentrations. For subsequent rounds, TAMR-Fc was used as soluble antigen, staining with an anti-human IgG PE-conjugate (1:50 dilution, Thermo Fisher Scientific).

### Next-Generation Sequencing

Plasmids were isolated from yeast cell populations of the initial library, as well as the 1st and 2nd sorting rounds using the Zymoprep Yeast Plasmid Miniprep Kit (Zymo Research). By means of PCR utilizing Q5 High-Fidelity DNA Polymerase (New England Biolabs), the VH and VL genes were amplified and purified by the PCR Clean-Up System (Promega). Next-generation sequencing by Illumina sequencing was performed at Genewiz (Leipzig, Germany) and resulting NGS data were analysed using Geneious Prime 2020.1.2.

### Reformatting

Plasmids of yeast cells, enriched during FACS, were isolated using the Zymoprep Yeast Plasmid Miniprep Kit (Zymo Research). Subsequently, plasmids were transformed into chemically competent XL1 blue *E. coli* cells and cultivated overnight in 50 ml dYT media with 100 μg/ml ampicillin. The next day, plasmids were isolated using the PureYield™ Plasmid Midiprep System (Promega). For reformatting into the mammalian expression vector, 75 ng of the YSD vector population, encoding the bidirectional CL-VL-Gal1,10-VH-CH1-partial hinge ORFs, was subjected to GGC with 75 ng of the Mammalian Destination (MD) vector, exhibiting the sequences encoding the partial Hinge-CH2-CH3. By *Esp*3I-mediated GGC, the complete bidirectional yeast-derived ORF cassette was inserted into the MD vector. While the 3’ end of the CL domain exhibited a stop codon, the CH1-partial hinge region of the yeast vector aligns with the partial-hinge-CH2-CH3 region of the MD vector, resulting in an ORF encoding the complete heavy chain. Subsequently, the Golden Gate products were transformed into chemically competent XL1 blue *E. coli* cells and cultivated overnight in 50 ml dYT media with 25 μg/ml chloramphenicol. Plasmids were isolated using the PureYield™ Plasmid Midiprep System (Promega) and 75 ng were subsequently subjected to GGC cloning utilizing the Mammalian_entry vector. By *Bbs*I-mediated GGC, the Gal1,10 promoter was removed from the vector and exchanged by the bidirectional 2xeCMV promoter cassette. The Golden Gate products were transformed into chemically competent XL1 blue *E. coli* cells and cultivated overnight in 50 ml dYT media with 25 μg/ml chloramphenicol and subsequently isolated by using the PureYield™ Plasmid Midiprep System (Promega). Golden Gate reactions were performed according to the manufacturer’s instruction. In brief, a 20 µl Golden Gate reaction containing 75 ng of the destination vector, 75 ng of the parental vector harbouring the desired insert, 2 µl T4 ligase buffer, 1 µl of the respective type IIS restriction enzyme (*Esp*3I or *Bbs*I), and 0.5 µl T4 ligase was used. All reagents for GGC reactions were supplied by New England Biolabs. Golden Gate cloning was performed in 30 cycles consisting each of 1 min at 37°C and 1 min at 16°C for restriction and ligation steps, respectively.

### Transient Transfection and Purification

Expi293-F cells (Thermo Fisher Scientific) were used for transient production of antibodies. The cells were cultured in Expi293 Expression Medium (Thermo Fisher Scientific) at 37°C, 8% CO_2_, and 110 rpm, and sub-passaged every 3–4 days. Transfection was carried out using Expifectamine293 according to the manufacturer’s manual, using 1 µg plasmid DNA per ml culture volume. Six days post-transfection, cells were harvested by centrifugation and the cell culture supernatants were sterile filtered before being applied to a HiTrap MabSelect PrismA column (Cytiva) using an ÄKTA Pure 25 system following the manufacturer’s instructions. Subsequently, a desalting step against PBS was performed using HiTrap Desalting columns (Cytiva).

### Biolayer Interferometry

For kinetics and affinity determination, an Octet Red96 (FortéBio) was utilised. Antibodies were loaded onto anti-human Fc Capture (AHC) biosensor tips (Sartorius) and associated to different concentrations of His-tagged TAMR in the range of 6.25–200 nM. Kinetics were determined using Savitzky-Golay filtering and fitted using a 1:1 Langmuir binding model.

### Thermal Stability and Size-Exclusion Chromatography

To evaluate the thermal stability of the isolated and reformatted antibodies, melting points (T_M_) were determined utilising the Prometheus NT.48 NanoDSF Protein Stability Instrument (NanoTemper Technologies) at 0.5 mg/ml between 25–90°C with a heating rate of 1°C/min as previously described (Bogen et al., 2020a). Size exclusion chromatography was carried out using an Agilent Technologies 1260 Infinity system as previously described (Bogen et al., 2020a). In short, 22 µl of a 0.5 mg/ml protein solution were applied onto a TSKgel SuperSW3000 column (Tosoh) at a constant flow rate of 0.35 ml/min using sterile filtered PBS (pH 7.4) as mobile phase.

## Results

### Overview of Cloning Strategy From Yeast-Surface Display to Mammalian Destination Vector

To ease the workflow from YSD-mediated antibody discovery to their expression in a mammalian system, we developed a high-throughput, bulk cloning procedure that avoids possible PCR-mediated mutations and conserves heavy chain—light chain pairing while yielding a full-length IgG molecule and significantly reducing hands-on time. In principle, VH and VL genes amplified from virtually any origin, for example, an immunised animal, can be subcloned by *Sap*I-mediated GGC into a YSD-vector utilizing the Gal1,10-based bidirectional promoter, similar to the previously described system (Rosowski et al., 2018). Upon transformation in *Saccharomyces cerevisiae* and FACS-assisted enrichment, yeast cells expressing target-specific Fabs are isolated. For subsequent characterization, expression of full-length IgG molecules in mammalian cells is crucial, as this represents the final expression system in the final format for therapeutic mAbs. However, bulk reformatting of multiple antibody candidates from an enriched pool of binders is not feasible with conventional methods as the original heavy chain—light chain pairing is lost during this process. This could be avoided by using scFvs or other single chain antibody formats. However, for most therapeutic applications, IgG antibodies are favoured as demonstrated by the number of approved IgGs in comparison to approved scFv-based drugs (Reichert, 2021). Furthermore, using Fab libraries for YSD allows for screening in an architecture most closely resembling the final IgG format and has shown to result in more promising antibody variants compared to scFv libraries (Sivelle et al., 2018).

Conventionally, the VH and VL sequences are reformatted for each single candidate separately in order to retain the heavy chain—light chain pairing. As more mAb candidates are available, this process becomes more laborious and tedious. Furthermore, PCR-based subcloning bears the risk of introducing unintended mutations, further increasing workload and slowing down the discovery process. To circumvent all these disadvantages, we engineered a mammalian destination vector (MD), encoding a partial hinge region followed by CH2- and CH3-encoding genes. By Golden Gate Cloning using *Esp*3I, the whole CL-VL-Gal1,10-VH-CH1-partial hinge ORF is extracted from the YSD vector and inserted into the MD vector, resulting in a full-length heavy chain ORF, enabling the transfer of a whole population of binding molecules in a one-pot reaction. Due to the physical linkage with the yeast Gal1,10 promoter, heavy chain—light chain coupling is maintained. In the next step, this yeast-specific promoter is exchanged by *Bbs*I-mediated Golden Gate Assembly for the 2xeCMV promoter, which was recently demonstrated to be the optimal bidirectional promoter for mAb expression (Carrara et al., 2021b). An overview of the entire procedure is depicted in Figure 1. This final vector enables transient transfection of mammalian cells for mAb production and additionally circumvents the need of co-transfecting separate heavy and light chain-encoding plasmids, which is needed in conventional procedures. All vectors were designed *in silico* and subsequently ordered at GeneArt (Thermo Fisher Scientific).

### PoC Study With Durvalumab

**FIGURE 2:**
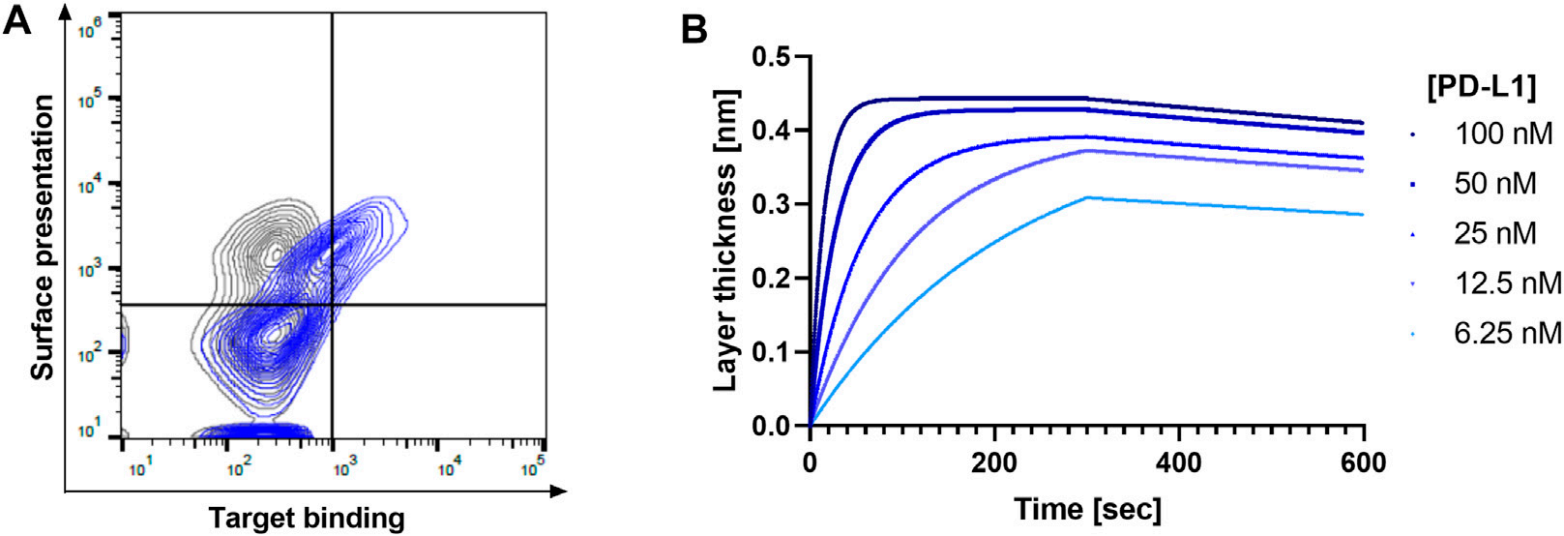
Verification of proof-of-concept study using Durvalumab. **(A)** FACS contour plot showing the display of the Durvalumab Fab and its ability to bind biotinylated PD-L1 (blue) compared to the negative control without antigen (grey). Surface presentation was detected using an anti-kappa AlexaFluor647 antibody, while target binding was detected using streptavidin-PE. **(B)** Affinity determination of Durvalumab to PD-L1 after production in Expi293-F cells. 10 μg/ml antibody was loaded and associated to different PD-L1 concentrations.

To verify whether this Golden Gate-assisted discovery process is feasible for subcloning of yeast-displayed Fabs into the mammalian expression vector, we performed a PoC study with the FDA-approved antibody Durvalumab, which recognizes the PD-L1 antigen and blocks the PD-1/PD-L1 axis. The respective VH and VL genes were subcloned into the yeast display vector and upon transformation of yeast cells and subsequent FACS analysis, the display of the respective Fab as well as PD-L1 recognition was verified (Figure 2A). Upon *Esp3*I-mediated subcloning of the antibody-encoding genes and the Gal1,10 promoter into the MD vector, followed by promoter exchange in a *Bbs*I-assisted manner, a mammalian expression vector was generated. Transient transfection of Expi293-F cells yielded full-length Durvalumab molecules able to recognize PD-L1 in BLI experiments (Figure 2B) with an affinity of 364 pM., similar to what has been described in literature (Tan et al., 2018). Encouraged by these initial results, we planned on translating this process to an antibody hit discovery campaign.

### Generation of TAMR Library and Screening

We initiated genetic immunisation of transgenic OmniRats, rodents exhibiting a part of the human antibody germline loci (Osborn et al., 2013), with TAMR. Upon cDNA synthesis after total RNA extraction of spleen and lymph node cells, VH- and VL-encoding genes were amplified and subcloned into the yeast display vector using *Sap*I-mediated GGC and the YSD_Entry_insert providing the bidirectional Gal1,10 promoter. For library generation, *Sap*I was chosen, as this type IIS restriction enzyme does not recognize any antibody germline sequences within OmniRat-derived mAbs. A summary of the cleavage sites within the OmniRat repertoire with the herein used type IIS restriction enzymes, as well as *Bsa*I as a reference, is represented in Supplementary Table S2.

**FIGURE 3:**
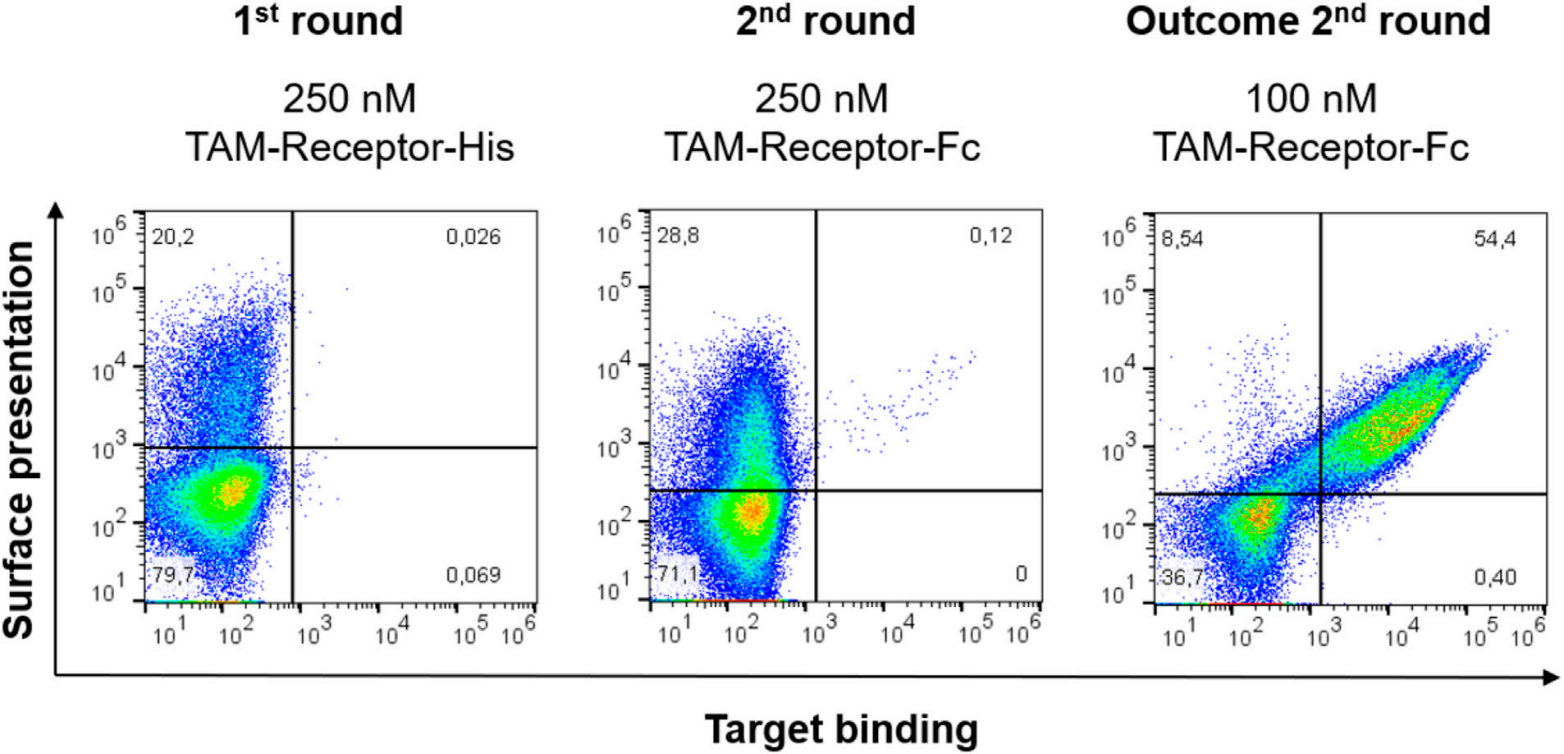
Sorting of the TAMR yeast library. Target binding to TAM-Receptor is depicted on the x-axis using either an anti-His AlexaFlour647-conjugated or anti-human IgG-PE conjugated antibody for either TAMR-His or TAMR-Fc, respectively. Surface presentation is shown on the y-axis as detected using a goat F (ab’)_2_ anti-human Kappa-AlexaFluor647 or -PE conjugate, respectively. 100’000 events are shown.

Upon yeast transformation, the resulting library consisted of 3 x 10^8^ clones and was screened *via* FACS. The first sorting round was performed using 250 nM His-tagged antigen. The subsequent screening round was performed with 250 nM Fc-tagged TAMR, and the outcome of round 2 was stained with 100 nM Fc-tagged TAMR to get higher affinity binders (Figure 3). A sufficient enrichment was observed after two sorting rounds, with 54.5% of the Fab-displaying yeast cells binding to the target antigen.

### Reformatting of YSD-Enriched Antibodies Into the Mammalian Destination Vector

The antibody-encoding plasmids from the yeast cells after the second sorting round were isolated and antibody-encoding genes were subcloned into the MD vector. Upon promoter exchange from Gal1,10 to 2xeCMV, the obtained final mammalian expression vectors were then subjected to Sanger sequencing. Analysis of 20 single clones revealed 14 unique antibodies, which, in part, originate from multiple clonotypes (Supplementary Figure S1). By clustering the sequences based on amino acid differences, especially in the CDR3, ten candidates were chosen for further analysis. These ten clones were referred to as K9, K12, K13, K15, K17, K22, K24, K26, K27, and K28.

### Next-Generation Sequencing

**FIGURE 4:**
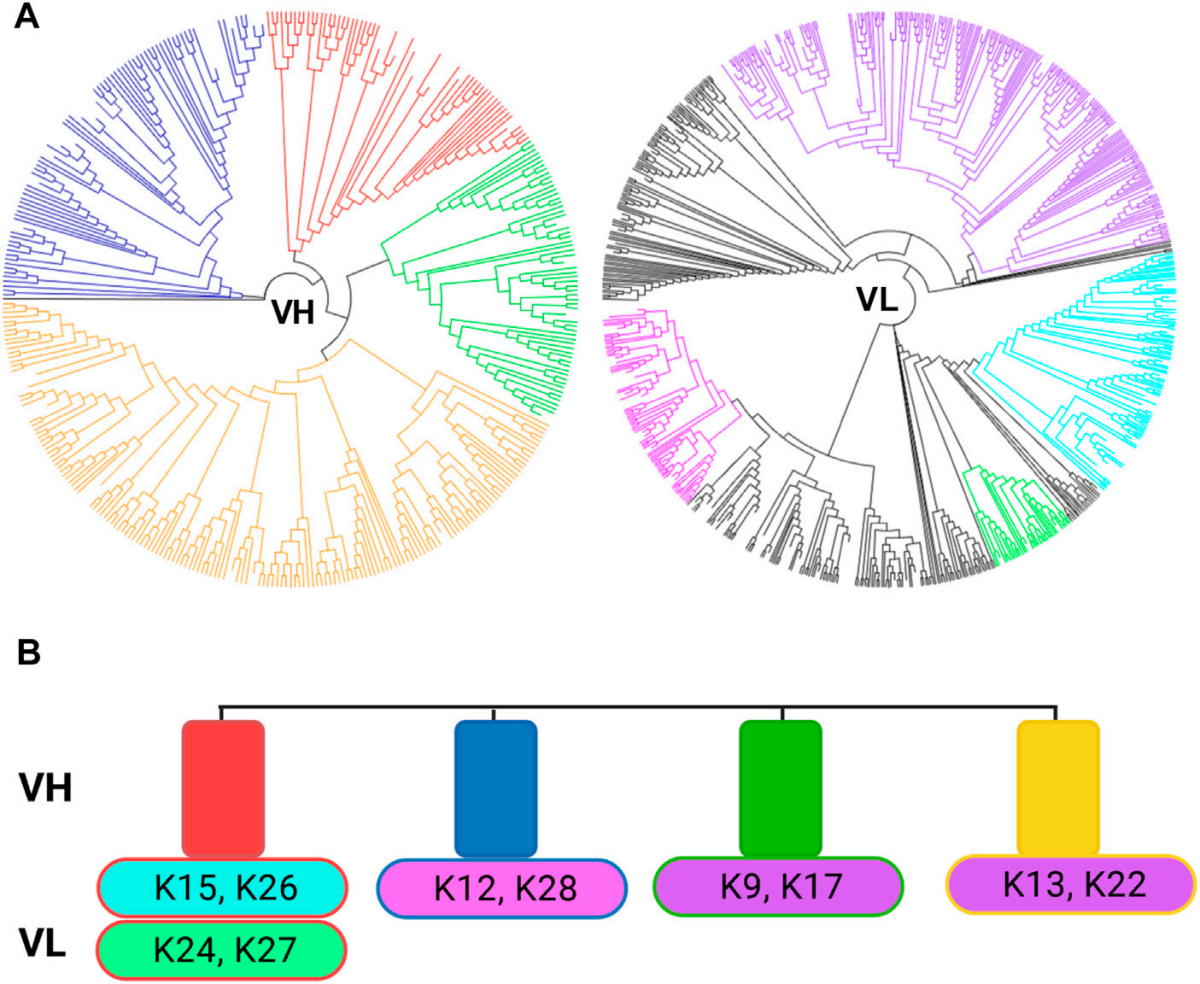
Next generation sequencing results from the 2^nd^ round of sorting. **(A)** Tree view of the VH and VL sequences after the second sorting round separated by VH and VL sequences. Tree maps were generated using Geneious Prime 2020.1.2. The colour-coding corresponds to the VH/VL sequences isolated in the 10 single clones depicted in B. **(B)** Representation of the found VH and VL in the 10 isolated clones. The rectangles represent the VH clusters, while the ovals represent the VL sequences. Colour-coding of the corresponding VH and VL pairs.

To confirm that no diversity is lost during the subcloning of the Fabs from the library to the expression vector, next-generation sequencing (NGS) was performed on the initial library as well as the 1st screening round outcome (Supplementary Figure S2). The library diversity from the last sorting round was compared to the individual sequences from isolated variants, and colour-coded with the respective sequences from isolated clones (Figure 4A). The entire diversity of VH sequence families obtained from library sorting was retained in the population of reformatted clones, whereas a few VL sequence families were missing. As only 20 clones were inspected, it is probable that more variability could be covered by sequencing a larger sample. Nonetheless, the VH diversity is generally sufficient to obtain highly specific antibodies (Xu and Davis, 2000) and thus is more critical. Figure 4B depicts the single clones and their respective VH and VL combinations. From this NGS analysis, one can appreciate that no large diversity was lost within the process as the entire VH repertoire was covered within the single clones.

### Transient Expression in Expi293-F Cells, Purification and Characterisation

**FIGURE 5:**
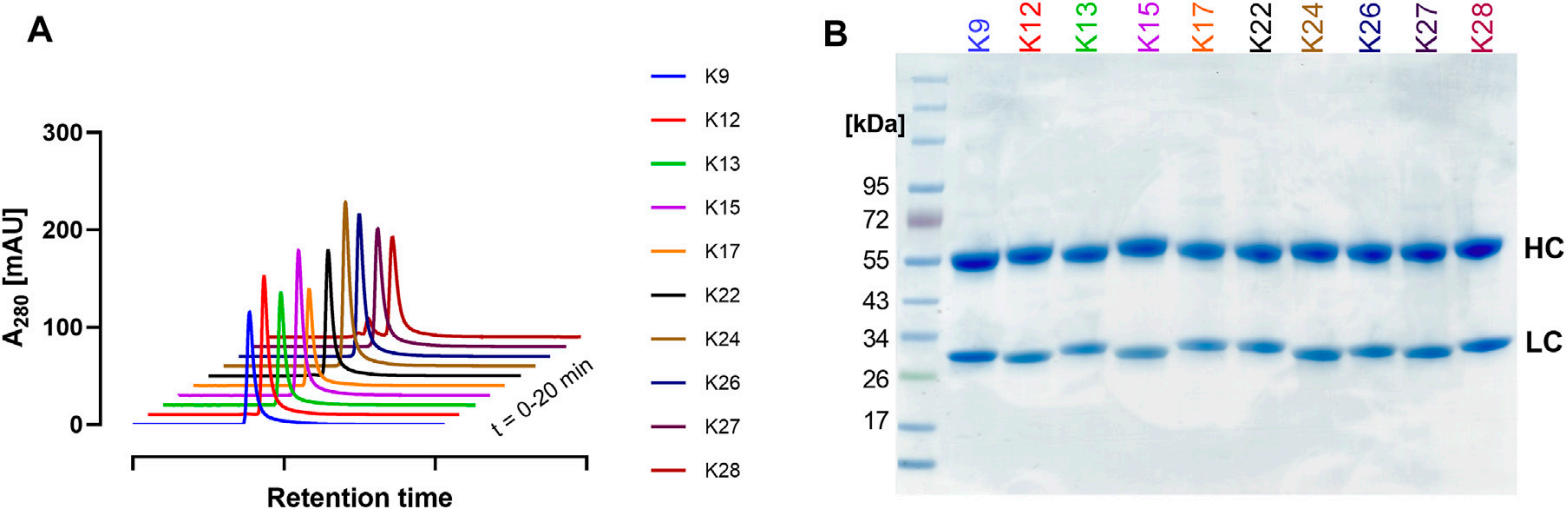
Characterisation of single clones. **(A)** SEC profiles and **(B)** SDS-PAGE analysis under reducing conditions, showing the respective heavy (HC) and light (LC) chains at their anticipated molecular weights. The curves in **(A)** are nudged by 1 and 10 data units in the x- and y-axis, respectively. Colour-coding represents the different single clones identified.

Ten single clones were produced in Expi293-F cells and purified *via* Protein A to characterise their biophysical properties. Size exclusion chromatography (SEC) revealed favourable aggregation behaviour for most clones, showing minimal aggregation (Figure 5A). K28 exhibited the most aggregation behaviour with 15.3% HMW. NanoDSF measurements showed melting temperatures (T_M_) between 65 and 70°C, with K13 and K28 exhibiting the lowest and highest T_M_ values with 65.7 and 70.8°C, respectively (Supplementary Figure S3). The observed T_M_s represent a high thermal stability and are within a reasonable range for non-glycosylated human IgG1 antibodies (Garber and Demarest, 2007; Dashivets et al., 2015). SDS-PAGE analysis revealed the expected heavy and light chain bands (Figure 5B).

Furthermore, kinetics determination *via* BLI indicated a range of single-digit nanomolar affinities to soluble His-tagged TAMR for the vast majority of clones, namely K9, K13, K15, K17, K22, K26, K27, and K28 (Supplementary Figure S4). K27 exhibited the highest affinity with 5.85 nM, whereas K9 showed the lowest affinity with 43.1 nM (Table 1). Nevertheless, the affinities are within a notable range.

**TABLE 1:** Affinities of isolated TAMR antibodies determined by BLI measurements using His_6_-TAMR.

Clone	Affinity ± S.D. (nM)
K9	43.10 ± 0.65
K13	9.81 ± 0.20
K15	8.23 ± 0.24
K17	10.10 ± 0.21
K22	12.50 ± 0.31
K24	11.90 ± 0.31
K26	7.94 ± 0.26
K27	5.85 ± 0.22
K28	11.10 ± 0.093

Taken together, the biophysical characterisation revealed 10 promising antibodies targeting TAMR in this proof-of-concept study using the novel approach in order to facilitate the transition from YSD to antibody production in mammalian cells.

## Discussion

In this study, we developed a straightforward reformatting approach to yield mammalian expression vectors based on YSD-screened antibody libraries in a PCR-free manner, based on Golden Gate Cloning. This process allows for bulk cloning of yeast display-enriched antibodies, which was previously not possible due to the loss of the heavy chain—light chain pairing, resulting in reduced hands-on time compared to conventional approaches. Although the issue of cognate heavy and light chain pairing can be omitted by the use of scFvs or scFabs, these molecules come with certain limitations, such as their aggregation behaviour (Röthlisberger et al., 2005; Bates and Power, 2019). Furthermore, the utilization of a Fab-displaying yeast library allows for screening in the final format of the antigen binding site for most therapeutic applications.

However, during library generation, VH and VL genes, amplified from cDNA isolated from immunised animals, are shuffled and therefore their natural pairing is lost. Still, this is also true for conventional yeast libraries, as well as for phage or mammalian display systems. Our novel system could be combined with B cell cloning in the future (Tiller, 2011; Carbonetti et al., 2017; Ouisse et al., 2017), which might allow for a workflow starting from B cells, followed by yeast display screening and mammalian antibody production while maintaining the naturally occurring heavy chain—light chain pairing during the whole antibody discovery process.

Previously, Roth and others have demonstrated the successful generation of a bidirectional YSD vector system to isolate CEACAM6-specific antibodies from immunised OmniRat libraries (Roth et al., 2018). For their library generation, *Bsa*I was utilized, which occurs frequently in antibody frameworks (Nelson and Valadon, 2017). To circumvent the risk of losing variants due to internal *Bsa*I sites, we chose *Sap*I for library generation, which does not occur in OmniRat-derived antibody germlines. As depicted in Supplementary Table S2, the other type IIS enzymes employed in this workflow do not have high occurrences within the OmniRat repertoire. Besides human antibodies, chicken-derived antibodies have gained traction within the scientific literature in recent years (Kim et al., 2018; Grzeschik et al., 2019; Bogen et al., 2020a; Bogen et al., 2020b; Bogen et al., 2020c; Hinz et al., 2020; Mwale et al., 2020; Elter et al., 2021). As the chicken germline does not exhibit *Sap*I sites either, this workflow can easily be applied to immunised chickens as well, and most likely, to other immunised animals, like mice or rabbits.

Future applications could also include antibody discovery workflows containing a combination of display technologies, for example, by cross-linking phage and yeast display and the presented MD vector for subsequent mammalian production (Patel et al., 2011). Additionally, Akamatsu and others have previously developed a mammalian cell surface display vector to isolate IgG molecules directly using mammalian display (Akamatsu et al., 2007) that can be used to combine yeast surface display with subsequent mammalian display and production. Such workflows can benefit from the advantages of each display technology but are not limited by their own disadvantages. For instance, combining the larger diversity of phage display libraries with the ability to sort for high-affinity antibodies (Marks, 2020) or to perform epitope binning by yeast surface display may ultimately reduce further screening steps within the discovery workflow (e.g., functional assays, binding affinities, etc.). The herein presented convenient and time-efficient two-pot, two-step method transition from YSD to mammalian expression vector can be further optimised to yield a one-pot, two-step reaction, performing the transfer of antibody VH and VL sequences as well as the promoter exchange within the same “pot”. Furthermore, improvement of the yeast preparation can obviate the need for transformation of *E. coli* and lead to the direct transition from the yeast preparation into the final MD vector for mammalian antibody production. Moreover, a toolbox of different MD vectors containing different Fc variants [e.g., LALA (Lund et al., 1991), N297Q (Wang et al., 2018), RF mutation (Jendeberg et al., 1997)] could be constructed to allow the practical and time-saving exchange for a whole population. This would also pave the way for establishing the methodology for bispecific or multi-specific antibody formats.

Taken together, we designed a straightforward method to preserve heavy chain—light chain paring while allowing bulk reformatting of YSD-enriched antibody hit candidates into mammalian expression vectors with reduced hands-on time. This procedure has the potential to speed up the drug discovery process and might aid the discovery of therapeutically interesting antibody candidates in the future.

## Data Availability

The original contributions presented in the study are included in the article/Supplementary Material, further inquiries can be directed to the corresponding author.
