# Supplementary material for: Streamlining the Transition From Yeast Surface Display of Antibody Fragment Immune Libraries to the Production as IgG Format in Mammalian Cells
Source: Front Bioeng Biotechnol. 2022 May 10;10:794389. doi: 10.3389/fbioe.2022.794389 (PMC9127228; doi:10.3389/fbioe.2022.794389)
Supplement: Supplementary file 1 [file DataSheet1.PDF]

Supplementary Material

1 Supplementary Figures and Tables

Supplementary Table 1: Primers for 2nd PCR with GGA SapI overhangs for VH and VL kappa

Primer Name	VH/VL	Sequence (5' - 3')
Omni_VH-GGA_1	VH	ATATATGCTCTTCAGCACAGGTBCAGCTGGTGCARTCTGG
Omni_VH-GGA_2	VH	ATATATGCTCTTCAGCACARRTSCAGCTGGTRCAGTCTGG
Omni_VH-GGA_3	VH	ATATATGCTCTTCAGCACAGRTCACCTTGAAGGAGTCTGG
Omni_VH-GGA_4	VH	ATATATGCTCTTCAGCASAGGTGCAGCTGGTGGAGTCYGG
Omni_VH-GGA_5	VH	ATATATGCTCTTCAGCAGARGTGCAGCTGKTGGAGTCTGG
Omni_VH-GGA_6	VH	ATATATGCTCTTCAGCACAGGTGCAGCTACAGCAGTGGGG
Omni_VH-GGA_7	VH	ATATATGCTCTTCAGCACAGSTGCAGCTGCAGGAGTCGGG
Omni_VH-GGA_8	VH	ATATATGCTCTTCAGCAGAGGTGCAGCTGGTGCAGTCTGG
Omni_VH-GGA_9	VH	ATATATGCTCTTCAGCACAGGTACAGCTGCAGCAGTCAGG
Omni_VH_rev_woEsp3I	VH	TATATATGCTCTTCTGGCTGARGAGACAGTGACCR
Omni_K-GGA_1	VL kappa	ATATATGCTCTTCAGCTGACATCCAGATGACCCAGTCTCC
Omni_K-GGA_2	VL kappa	ATATATGCTCTTCAGCTGMCATCCRGWTGACCCAGTCTCC
Omni_K-GGA_3	VL kappa	ATATATGCTCTTCAGCTGATRTTGTGATGACYCAGWCTCC
Omni_K-GGA_4	VL kappa	ATATATGCTCTTCAGCTGAAATWGTGWTGACRCAGTCTCC
Omni_K-GGA_5	VL kappa	ATATATGCTCTTCAGCTGACATCGTGATGACCCAGTCTCC
Omni_K-GGA_6	VL kappa	ATATATGCTCTTCAGCTGAAACGACACTCACGCAGTCTCC
Omni_K-GGA_7	VL kappa	ATATATGCTCTTCAGCTGAAATTGTGCTGACTCAGTCTCC
Omni_K-GGA_rev1	VL kappa	GCGCGCGCTCTTCATCGTTTGATHTCCASYTTGGTCCC
Omni_K-GGA_rev2	VL kappa	GCGCGCGCTCTTCATCGTTTAATCTCCAGTCGTGTCCC

Overhang sequence

Primer sequence

Supplementary Table 2: Number of cleavage sites in the OmniRat repertoire. A total of 44 VH and 20 Vk germline genes are present. *Bsa*I serves as a reference, compared to the three type IIS enzymes used to establish the workflow presented in this work.

**Type IIS Enzyme**	**Number of cleavage sites in OmniRat germlines**	
	**VH**	**Vk**
*Sap*I	0	0
*Bbs*I	2	0
*Esp*3I	1	0
*Bsa*I	15	0

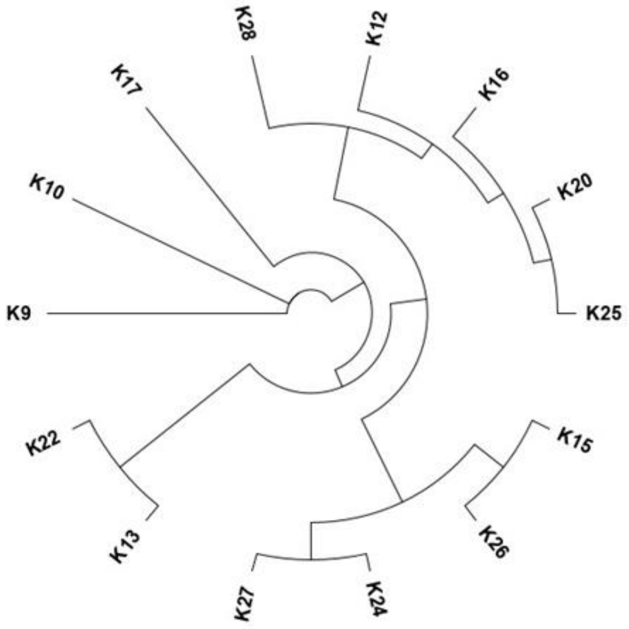

Supplementary Figure 1: Tree map of VH-VL pairs after reformatting into the MD vector. 14 unique candidates based on sequence differences were revealed.

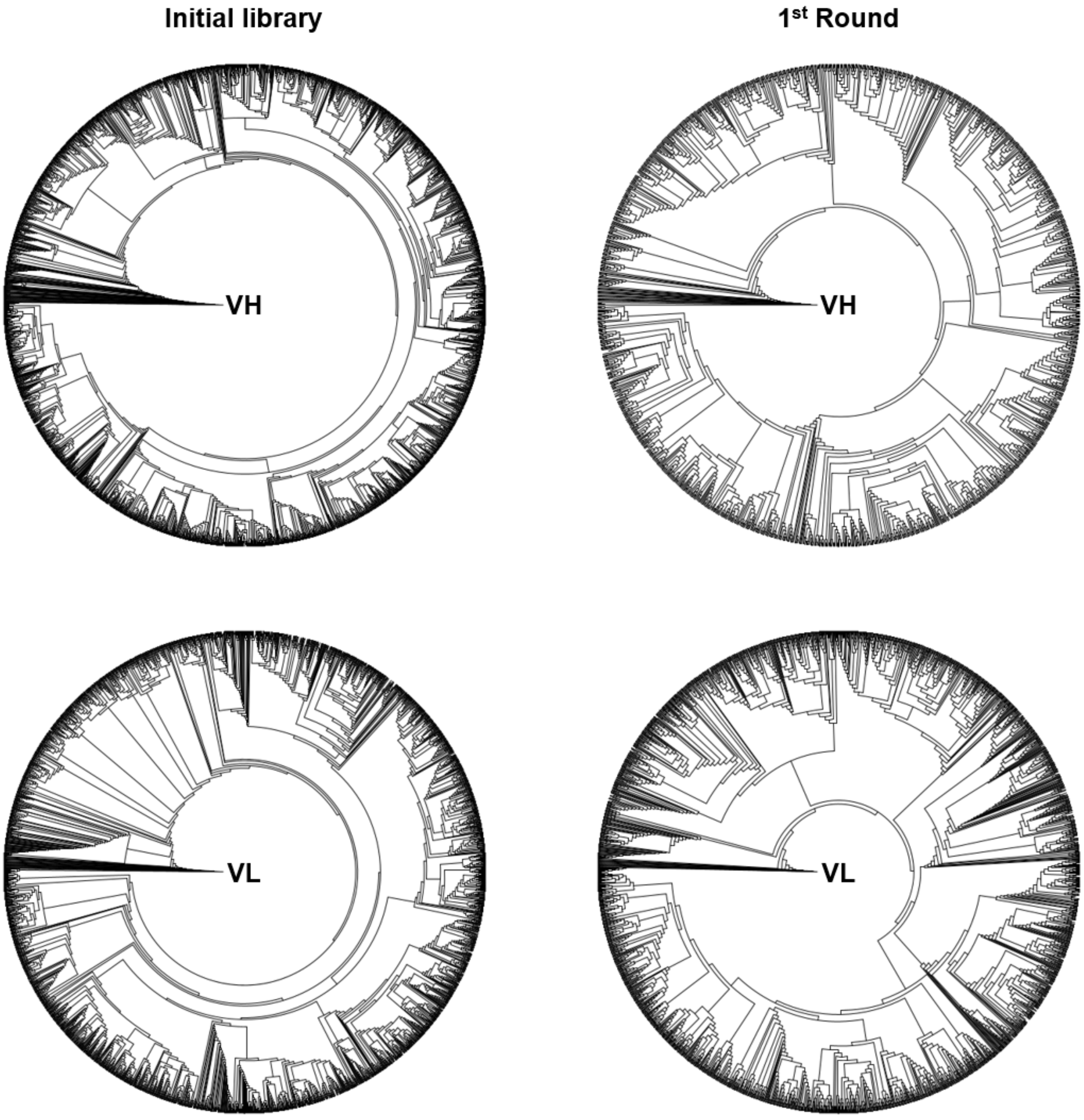

Supplementary Figure 2: VH and VL diversity of the initial library and after the 1st screening round on the left and right, respectively, after NGS analysis.

Clone	T_M [°C]
K9	68.1
K13	65.7
K15	69.0
K17	68.6
K22	70.3
K26	70.5
K27	68.5
K28	70.8

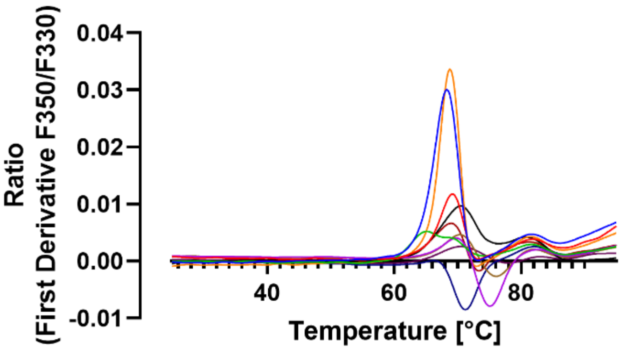

Supplementary Figure 3: Melting temperatures of TAMR-binding variants. NanoDSF-assisted thermal stability studies were performed. The ratio of the integrated fluorescence at 350 nm / 330 nm was calculated. Colour-coding of the clone names correlates with the curves.

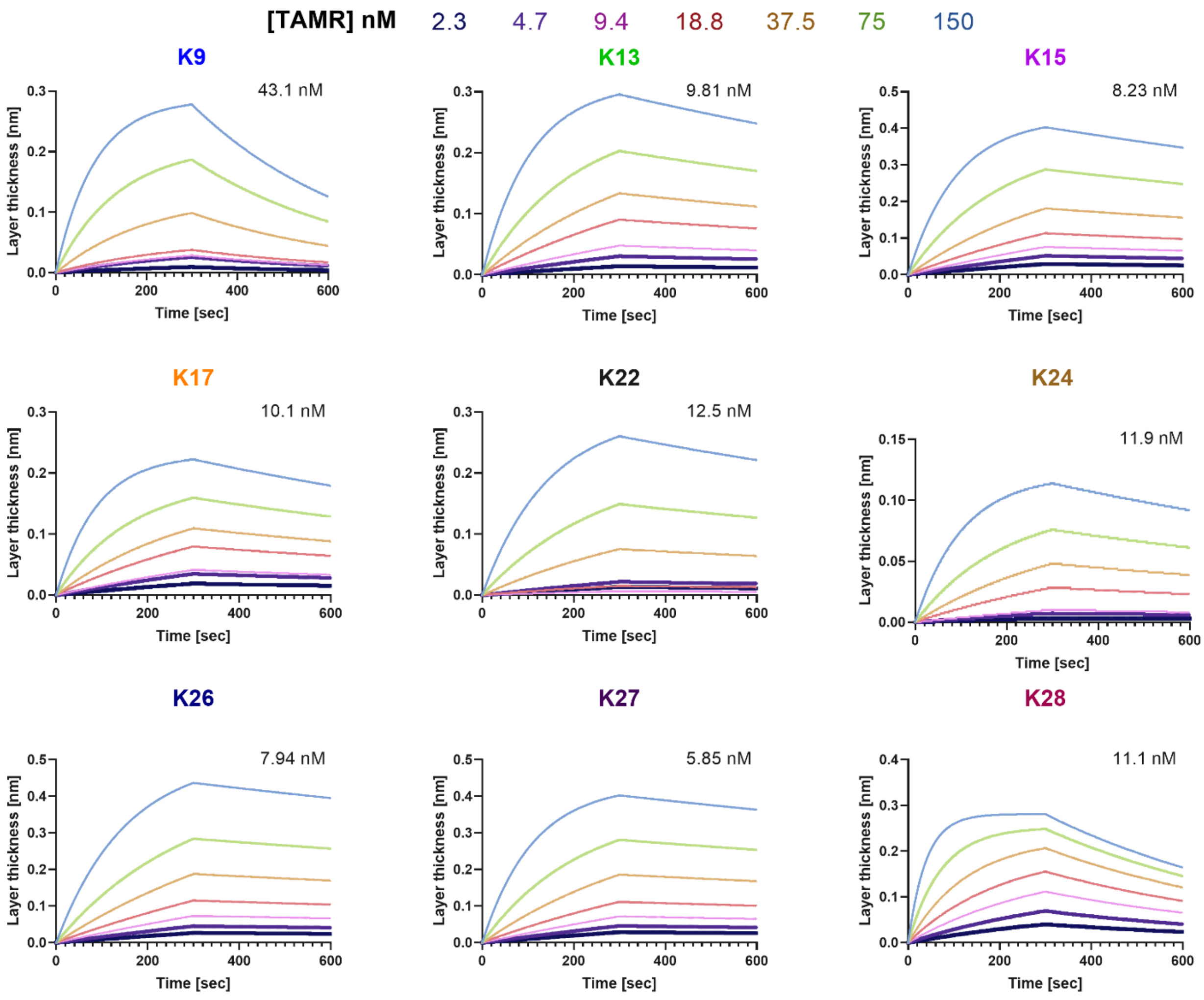

Supplementary Figure 4: Kinetics determination of variants targeting TAMR. Antibodies were immobilized at a concentration of 10 µg/ml on AHC biosensor tips and associated to different concentrations of soluble antigen in a range from 0 – 150 nM.
